# "Aseptic" and "Antiseptic" Surgery

**Published:** 1902-12-20

**Authors:** 


### "Aseptic" and "Antiseptic" Surgery.

In the *British Medical Journal* of last week a large amount of space is devoted to an account of Lord Lister's great discoveries and to well-deserved praises of the man himself. To the details there given we need not refer ; they are more or less historical and accepted of all men. It is interesting to note, however, that among the various articles on the subject a note of doubt is sounded by several writers as to the recent more extreme developments of what is called "aseptic surgery" in contradistinction to that system which involves the moderate use of antiseptics. Dr. Lucas-Championnière points out that from the beginning Lister laid down a principle which many surgeons have too often forgotten, namely, that "an antiseptic is injurious to the cellular elements of the body as well as to the microbes. The art of the surgeon lies therefore in employing it in a sufficient but not an excessive dose." People now, however, go far beyond this, and "we find ourselves face to face with an entire school which maintains that the surgery of Lister is at an end, that it has had its day, and that progress has swept it away," and Dr. Lucas-Championnière asks, "Is it possible to abolish all antiseptics and to say, as has been noisily said, that the method of Lister has been abandoned, antisepsis replaced by asepsis?" and he answers "By no means." Dr. Lucas-Championnière points to the elaborate precautions necessary for the safe conduct of aseptic work compared with the independence of his surroundings enjoyed by the surgeon who makes use of antiseptics. Aseptic surgery "is laboratory work. Never before have the architects been more indispensable. The least defect in the condition of an operation-room manifests itself by disasters. If the surgeon do not surround himself with impracticable precautions—masks, gloves, etc.—the safety of the patient is imperilled. . . . And yet have they altered the results of surgery? Certainly they have caused it to lose its safety.

In contrast with this he speaks of antiseptic surgery. "The environment may be bad, the hospital may be crowded, contagion near at hand, the operating-room may be too small or too large ; all these things are of small account. The patient will recover because the surgeon will be master of the field of operation. This field is quite small. It can be efficaciously protected. Prudence demands that the badness of the conditions under which operations are performed shall not be exaggerated. An evil environment must not be created, but if it exists it can be defied." Evidently Dr. Lucas-Championnière regards "the failure of the modern method without antiseptics" as a fact incontrovertible. We fancy, however, there will be many who will join issue with him in regard to this. Indeed, unless the reports of hospital work done not only in Germany and America, but also in this country, are to be regarded as untrustworthy—which we do not for a moment believe—it must be admitted that aseptic surgery (by which we mean the entire abolition of the application of chemical antiseptics to clean wounds and their absence from all dressings placed upon such wounds) has attained to a great success. So far as hospitals are concerned we are never likely to go back from the architectural and physical means now employed in the promotion of cleanliness. At the same time it must be borne in mind that the success of "aseptic" surgery, according to the present usage of the phrase, depends upon a long chain of circumstances, upon the maintenance of the strictest discipline among a considerable number of assistants, upon the absolute honesty of a considerable number of purveyors of materials, and upon the structural perfection of many of the architectural and other details of the operating-room ; and it has to be confessed that this chain of circumstances is no stronger than its weakest link. An error by a nurse, the folly of some scrubber, a trumpery bit of economy on the part of a storekeeper, a breach of discipline anywhere along the line, may lead to failure ; and this being the case we can hardly wonder that, among those who do not care to delegate responsibility, a certain degree of reaction should arise against the growing custom of placing implicit, and as some would have it overweening confidence in a purely "aseptic" surgical routine.

